# Supplementary material for: The Systems Biology Research Tool: evolvable open-source software
Source: BMC Syst Biol. 2008 Jun 29;2:55. doi: 10.1186/1752-0509-2-55 (PMC2446383; doi:10.1186/1752-0509-2-55)
Supplement: Additional file 1 — SBRT Archive. An archive of the current version of the Systems Biology Research Tool. [file 1752-0509-2-55-S1.zip › sbrt-1.4.0/doc/developers_guide/api/sbrt/kernel/math/graph_theory/DirectedGraph.Edge.html]

DirectedGraph.Edge


|  |  |  |
| --- | --- | --- |
| **Overview** \| **Package** \| **Class** \| **Use** \| **Tree** \| **Deprecated** \| **Index** \| **Help** | |  |
| **PREV CLASS**   **NEXT CLASS** | **FRAMES**    **NO FRAMES**     **All Classes** |  |
| SUMMARY: NESTED \| FIELD \| CONSTR \| METHOD | DETAIL: FIELD \| CONSTR \| METHOD |  |


---


## sbrt.kernel.math.graph\_theory Class DirectedGraph.Edge<N>

```
java.lang.Object
  sbrt.kernel.math.graph_theory.DirectedGraph.Edge<N>
```

**Type Parameters:**: `N` - the type of node.

**Enclosing class:**: DirectedGraph<N>

---

``` public static final class DirectedGraph.Edge<N> extends java.lang.Object ```

This class is used to represent the unidirectional
edges of a directed graph. An edge is directed from a
source node to a sink node.

**Author:**
:   This class was written and documented by
    Jeremiah Wright while in the Wagner lab.

---

**Constructor Summary**

`DirectedGraph.Edge(T source,
S sink)`
  
          Constructs a new directed edge from the provided
source and sink nodes.


| **Method Summary** | |
| --- | --- |
| `boolean` | `equals(java.lang.Object o)`             Indicates if the provided object is equal to this directed edge. |
| `N` | `getSink()`             Returns the sink node of this directed edge. |
| `N` | `getSource()`             Returns the source node of this directed edge. |
| `int` | `hashCode()`             Returns a content-based hash code for this directed edge. |
| `java.lang.String` | `toString()`             Returns an appropriate string representation of this edge. |

**Methods inherited from class java.lang.Object**

`clone, finalize, getClass, notify, notifyAll, wait, wait, wait`

**Constructor Detail**

### DirectedGraph.Edge

```
public DirectedGraph.Edge(T source,
                          S sink)
```

:   Constructs a new directed edge from the provided
    source and sink nodes.

    **Type Parameters:**: `T` - a type that extends N.: `S` - a type that extends N. **Parameters:**: `source` - the source node.: `sink` - the sink node.


**Method Detail**

### getSource

```
public N getSource()
```

:   Returns the source node of this directed edge.

    :   **Returns:**: the source node of this directed edge.

---


### getSink

```
public N getSink()
```

:   Returns the sink node of this directed edge.

    :   **Returns:**: the sink node of this directed edge.

---


### equals

```
public boolean equals(java.lang.Object o)
```

:   Indicates if the provided object is equal to this
    directed edge. An object is equal to this edge if
    1) it is an instance of `Edge`, 2)
    its source node is the same as this edge's, and
    3) its sink node is the same as this edge's.

    :   **Overrides:**: `equals` in class `java.lang.Object`
    :   **Parameters:**: `o` - the reference object with which to compare. **Returns:**: `true` if this object is equal to the provided object; `false` otherwise.

---


### hashCode

```
public int hashCode()
```

:   Returns a content-based hash code for this directed edge.

    :   **Overrides:**: `hashCode` in class `java.lang.Object`
    :   **Returns:**: a content-based hash code for this directed edge.

---


### toString

```
public java.lang.String toString()
```

:   Returns an appropriate string representation of this edge.

    :   **Overrides:**: `toString` in class `java.lang.Object`
    :   **Returns:**: an appropriate string representation of this edge.


---


|  |  |  |
| --- | --- | --- |
| **Overview** \| **Package** \| **Class** \| **Use** \| **Tree** \| **Deprecated** \| **Index** \| **Help** | |  |
| **PREV CLASS**   **NEXT CLASS** | **FRAMES**    **NO FRAMES**     **All Classes** |  |
| SUMMARY: NESTED \| FIELD \| CONSTR \| METHOD | DETAIL: FIELD \| CONSTR \| METHOD |  |


---
